# Supplementary material for: Exploring virulence and stress response in Entamoeba histolytica: insights from clinical strains
Source: Microbiol Spectr. 2025 Jun 9;13(7):e00506-25. doi: 10.1128/spectrum.00506-25 (PMC12210921; doi:10.1128/spectrum.00506-25)
Supplement: Supplemental figures — Fig. S1 and S2. [file spectrum.00506-25-s0001.pdf]

1A. U-2 (EHI_C00154)

```
Entamoeba histolytica    1   MSSSKDKQTKSKIALIGGAVVTAVVAVGASTGAAVASLTDHANCTDRPFGSGATDFSWMS  60
Entamoeba nuttalli       1   MGSSKDKQTKSKIAIIGGAIATAVVGVGASTGAAVASLTDHANCTDRHFGSGATDFSWMS  60

Entamoeba histolytica    61  ENSSDY  66
Entamoeba nuttalli       61  ESSSD   65
```

1B. U-15 (EHI_124550)

```
Entamoeba histolytica   1     MKDGYKTKRYSIGIKCPSSLNPEWEDISHTIQQRVQKLNKKFHKDIKIELVQPSQVDHII  60
Entamoeba nuttalli P19  1     MKDDYKTKRYSIGIKCPSSLNPEWEDISHTIQQRVQKLNKKFHKDIKVELVQPSQVDHII  60
Entamoeba dispar SAW760 14    MKDDYKTKRYSIGLKCPPALNPEWGDISHSIQQRVQKLNKKFHKDIKVNLVQPNQVDHII  73
Entamoeba invadens IP1  20           KRFSISVFCPEGKDEEWNNINTTVQQRVKKLNKKFHKDIAVDVVKTNVADKTV  72

Entamoeba histolytica   61    EQSESEEVIIENNSNERRTTRRERRRKRKTRKVQLQANQEPRVNHTHQRIITIRNRQELE  120
Entamoeba nuttalli P19  61    EQSESEEVIIENNNNERRTTRRERRRKRKTRKVQLQANQEPRVNRTHQRIITIRNRQELE  120
Entamoeba dispar SAW760 74    EQSESEEVIIENNNNGMRTTRRERRRKRKTRRVQLQANQEPRVNRTHQRIVTIRNRQQLE  133
Entamoeba invadens IP1  73    DESASEEVHMDAQPQKR---RKQTHRERRLRRRLLEANQEPRVHHRH--YFRIRNNEQLQ  128

Entamoeba histolytica   121   AYLLSIIYIIFVCCVSLLIAFVIIIILI  148
Entamoeba nuttalli P19  121   AYLLSIIYIIFVCCVSLLIAFVIIIILI  148
Entamoeba dispar SAW760 134   AYLLSIIYIIFVCCVS              149
Entamoeba invadens IP1  129   AALLLVLYFIFLFCVSAIITLVVLILLI  156
```

1C. U-16 (EHI_107170)

```
Entamoeba histolytica   1     MIVFILLILISEAIETKLFKEQRVKDDKECQNLRVTTHTITNNINKSKNNDFSQEVEEDI  60
Entamoeba nuttalli P19  1     MITFILFVVFTTATYNVAHDDQGVKDVNKYQNI-VLFSKINRNIDKSKNNKYYIKYEEVF  95

Entamoeba histolytica   61    RVSKSNVFRTSKNLKHGTITTEDIEAAENYFSTGDTKRQYASDNILLKVEEAKPTPQEKP  120
Entamoeba nuttalli P19  96    RINKNIISTTSKNLKHGTVTSEDIENAENYFYYTNTKRMFNVYNIHLKGE----------  145

Entamoeba histolytica   121   SVTATNTHEEEKSKGNTEGGAQNEATTKNSETGESKKQEGSGNGQTSEGENKAIKGTEVS  180
Entamoeba nuttalli P19  146   -----NEHKESQS-AEVSGGEESSKKSHESETSQKAQSNSQSPSTTVTSPTTSEQGTQTS  200

Entamoeba histolytica   181   GGGSSSNQKVSSGENSAETGAPSASGSATTGKGGKGSESEHTVESGTSGQGGSETTSEGS  240
Entamoeba nuttalli P19  201   ESGAKEQQKVNEGQNQEGVGKNSLDGTSATGPGVSGQEATQTGEPSTGTQEGNTAVNKKP  283

Entamoeba histolytica   241   KVSEKTDVKNQESGGSQTVGQEGQGQKENGETGGKTDKGSSGTSSSQTQEQNSQNINGKG  300
Entamoeba nuttalli P19  284   EATESTNGGNTESGKS---GSEKNGQDQ--------------SADEKTKEEPKSN-----  321

Entamoeba histolytica   301   EVGGSDSSGKSGKEGQPSGGEKGDSGQEAGKEQKEQAGNEGATGEGKKESDSGVAGEGQK  360
Entamoeba nuttalli P19  322   --DGKDSTTSNGKGGQASGKEEETSGQSSGK------GGESTSNESGKENEESVGPNGQK  375

Entamoeba histolytica   361   ENENKGSVDDNKSDSKGGDGEKGNDGVAGNQAKDNKETEGQTDNNEQNNQQQNEQNNTKV  420
Entamoeba nuttalli P19  376   TPANEKDGKDTQT------GEQGKDGNAGTNGKDEKE-------NKQNTQTQDGKANDKT  428

Entamoeba histolytica   421   DNNEEHKDNKTTNTSESKPDNKTDVDSTSYTNLDSSSSVFLILSITLTFLFF  472
Entamoeba nuttalli P19  432   DNTKDNQDPKTPVTPEEKPENKNDDSSNNHNNLDSASSVCIFLSIIIGFLLF  501
```

Figure S1. Protein sequence alignment of four selected genes from Entamoeba species and other organisms. (1A) U2 (EHI_C00154) sequence alignment between *E. histolytica* and *E. nuttalli*. (1B) U15 (EHI_124550) sequence alignment between *E. histolytica* and other *Entamoeba* species. (1C) U16 (EHI_107170) sequence alignment between *E. histolytica* and *E. nuttalli*. (1D) U18 (EHI_176850) sequence alignment among *E. histolytica*, other *Entamoeba* species, and additional organisms. Yellow boxes indicate fully conserved residues across all organisms, while blue boxes highlight fully conserved residues only within *Entamoeba* species.

1D. U-18 (EHI_176850)

```
Entamoeba histolytica      1    MSEDTSPINEIEQMKKEYEEKIKKMTEENKKQIEEVKRLKGLILKQEEAQTEIVSGYEEQ  60
Entamoeba nuttalli P19     1    MSEDTSPINEIEQIKKEYEEKIKKMTEENKKQIEEVKRLKGLILKQEEAQTEIVSGYEEQ  60
Entamoeba dispar           1    MSVDTSPINEIEQIKKEYEEKIKEMTNENKKQIEEVKRLKGLILKQEEAQTEIVSGYEEQ  60
Entamoeba invadens IP1     6                                LCNELKKQQDEVKRLKLLILKQEDSQTEIVSSYEEQ  41
Solanum pennellii          393                                 DEDSKIIEELRQQRAQILQLEKALKQAVAG-QED  429
Sphagnum jensenii          387  MTEEIKAAEEACSMRAEFEHKLEEVSLERDKALRDLSRLKQSLLDMDMSNSEKMDQDREQ  447
Adiantum capillus-veneris  296                  KNYEKKLAAACAERDKAVRDLKRLKQHLLEKEQADSEMVRALEQN  361

Entamoeba histolytica      61   ISQLQKNTQVIEKQKEEIEEVKQENKELQKSIERANTEIEKQKKIIQTLSGIGRKMKEDQ  120
Entamoeba nuttalli P19     61   ISQLQKNTQVIEKQKEEIEELKQENKELQKSIERVNTEIEKQKKIIQTLSGIGRKMKEDQ  120
Entamoeba dispar           61   ISRLQKNTQIIEKQKEEIEELKQENKELQKSIEIANNEIEKQKKIIQTLSGIGRKMKEDQ  120
Entamoeba invadens IP1     42   IEKLKSDISLIPKQTEQIKTLTLQVAQLQETISKQTTDIQNKQRIIQTLSGIGRKMKEEQ  101
Solanum pennellii          430  VKTLNYNE--LRKSKETIDEL---NKRLASCL---NTMEAQNIEVLNLQTALGQYYAEIE  481
Sphagnum jensenii          448  IAELKVRTEVSEQLQQALNQAHTELAEVTKSLQQSSKEIQKLASCIDALSALGQYYAESQ  532
Adiantum capillus-veneris  362  LAQLQKQAEVNKRFNERLEEANQEIASLRKKLAACLNASESKDSELQNLAALGQYYAEGE  423

Entamoeba histolytica      121  EKIIKEKVTILEEELEQIKKENQRKVKENEELNTEIKDITNQLKEMDEIKQLNIQRNETI  180
Entamoeba nuttalli P19     121  EKIIKEKVTILEDELEQIKKENQRKVKENEELNTEIKDITNQLKEMDEIKQLNIQRNETI  180
Entamoeba dispar           121  EKIIKEKVTILEEELEQTKKENQRKAKENEELNTQIKDITNQLKEMDEIKQLNVQRNETI  180
Entamoeba invadens IP1     102  DTIFKEKMDVLNEELVASQKATVDAQNVNKALNKQIDEFKKTVLELEELKTTNAQRNETI  161
Solanum pennellii          482  AKRLGEELVMAKEELHKMSGLLKDAYNESETLKKEKEEVLVKLSDME--RRLSEGKGR-I  539
Sphagnum jensenii          533  ERLFGE-LTAAKEEISKLSEDLSIANKAIEMKNKEINEAVEKLK-MAELRKQEWEQNSR-  589
Adiantum capillus-veneris  424  AKRIQTELSAARKELEQLKSANNAIMVKDGEKGELLEKLALQQKKVSESQ-------EQS  481

Entamoeba histolytica      181  TKLYEENETLKKTVENFTQDLQSKIMNETYYVDKRIVNKLLISYITKPHQRMEIVNLMSK  240
Entamoeba nuttalli P19     181  TKLYEENETLKKTVENFTQDLQSKIMNETYYVDKRIVNKLLISYITKPHQRMEIVNLMSK  240
Entamoeba dispar           181  TKLYEENETLKKTVENFTQDLQNKIMNETYYVDKRIINKLLISYITKPHQRMEIVNLMSK  240
Entamoeba invadens IP1     162  TKLYEENETLKKAVEHFTQDMQSRIMNESFYVDKRIVNKLLISYITKPHQRTEIVELMSK  221
Solanum pennellii          540  SKLEQDNEKLRRAVEQSMTRLNRMSLDSDNYVDRRIVIKLLVTYFQRNHSK-EVLDLMVR  598
Sphagnum jensenii          590  -KLEEDVPRLRQALEQSITRLNRMSSDSDFYVDRRIVIKLLVTYFQRQHSR-EVLDLMVR  647
Adiantum capillus-veneris  482  HKLEADVLMLRRALEQSLTRLNRMSSDSDYYVDRRIVIKLLVTYFQRQQSR-EVLDLMSR  540

Entamoeba histolytica      241  IMDFTDEEKRLLGLSQETTQTGLFNYFFGKTDEYDEPPKYDLKDKTFGDLWVEFLLRESG  300
Entamoeba nuttalli P19     241  IMDFTEEEKRLLGLSQETTQTGLFNYFFGKTDEYDEPPKYDLKDKTFGDLWVEFLLRESG  300
Entamoeba dispar           241  IMDFTEEEKRLLGLSQETTQTGLFSYFFGKTDEYDEPPKYDLKDKTFGDLWVEFLLRESG  300
Entamoeba invadens IP1     222  IMDFTQEEKQVLGVAKETESKGIFSYFFGKTDEYEQAPKYDIKDKTFGDLWVEFLLRESG  281
Solanum pennellii          599  MLGFSDEDKQRIGMAQQGSGKGVVRGVFGLPGRLSSVPSTTASDQSFADLWVDFLLKEN   664
Sphagnum jensenii          648  MLGFTEEDKQRVGLAQQTTKGGVVRGVFSLPGRFDASPRAAPSDQSFADLWIDFLL      714
Adiantum capillus-veneris  541  MLGFSEEDKQVIGLAQQSSSKGVVRGVLGLPGRVSSPSVMSLSNQSFSDLWIDFLLKES   610

Entamoeba histolytica      301  SLDAQQPTNQK  311
Entamoeba nuttalli P19     301  SLDAQQPTNQK  311
Entamoeba dispar           301  SLDAQKPTNQK  311
Entamoeba invadens IP1     282  SLDS         285
```

Figure S1. Protein sequence alignment of four selected genes from Entamoeba species and other organisms. (1A) U2 (EHI_C00154) sequence alignment between *E. histolytica* and *E. nuttalli*. (1B) U15 (EHI_124550) sequence alignment between *E. histolytica* and other *Entamoeba* species. (1C) U16 (EHI_107170) sequence alignment between *E. histolytica* and *E. nuttalli*. (1D) U18 (EHI_176850) sequence alignment among *E. histolytica*, other *Entamoeba* species, and additional organisms. Yellow boxes indicate fully conserved residues across all organisms, while blue boxes highlight fully conserved residues only within *Entamoeba* species.

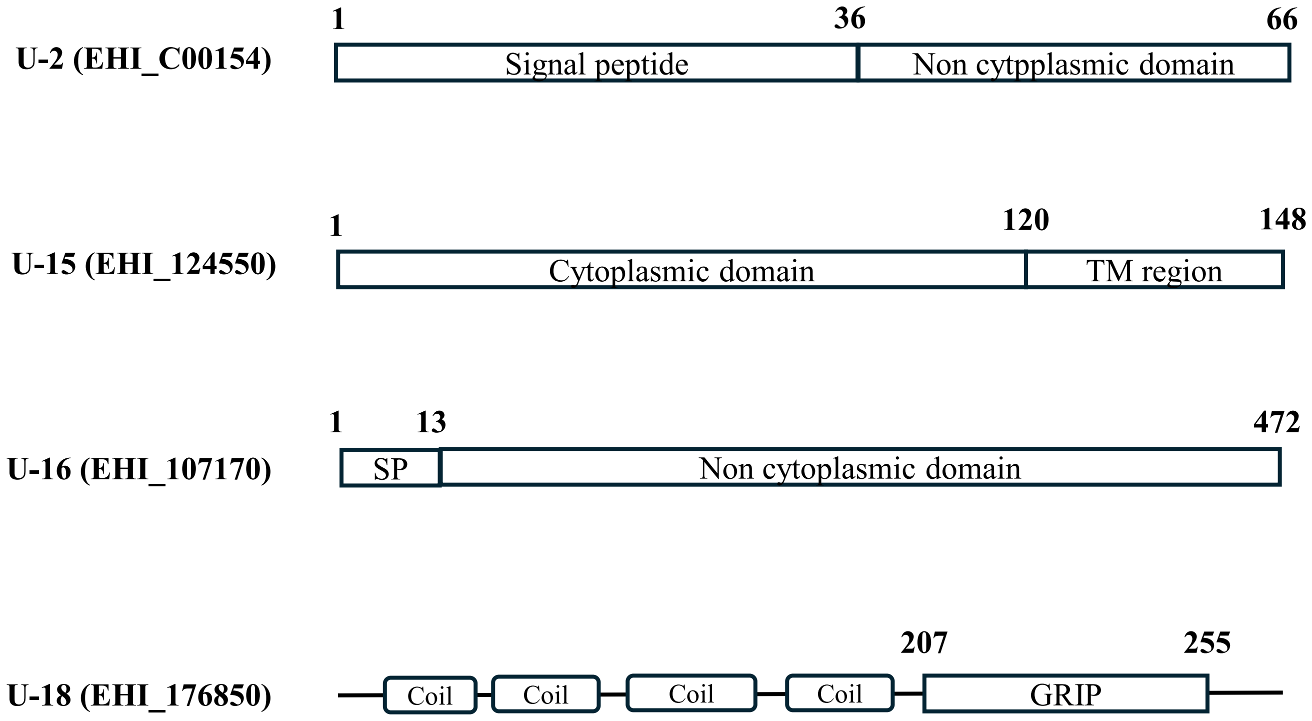

Figure S2. Diagrammatic representation of the domain architecture of four target genes. According to the InterPro predictions, each gene encodes translated sequences corresponding to specific domains and signal peptides. The domain abbreviations used in the diagram are as follows: TM, transmembrane; SP, signal peptide; GRIP, golgin-97, RanBP2alpha, Imh1p and p230/golgin-245.
