# Supplementary material for: Exploring virulence and stress response in Entamoeba histolytica: insights from clinical strains
Source: Microbiol Spectr. 2025 Jun 9;13(7):e00506-25. doi: 10.1128/spectrum.00506-25 (PMC12210921; doi:10.1128/spectrum.00506-25)
Supplement: Supplemental tables — Tables S1 and S2. [file spectrum.00506-25-s0002.pdf]

Supplementary Table 1. Primer sequence for RT-PCR

Research ID	Gene ID	Forward primer	Reverse primer
U2	EHI_C00154	AGCTCTAATTGGAGGTGCAG	AGAATTTTCCGACATCCATGA
U15	EHI_124550	TTCAGGCAAATCAAGAACCTC	TCAGATCAAAATGATAATAATGACAAA
U16	EHI_107170	GTAGTTCTCAAACACAAGAGC	AGTGTAATAGAAAGAATAAGAAATA
U18	EHI_176850	ACCACATCAACGAATGGAAAT	TGATTTGTTGGTTGTTGAGCA

Supplementary Table 2. Primer sequence for plasmid construct design

Research ID	Gene ID	Forward primer	Reverse primer
U2	EHI_C00154	CCCGGGATGAGTTCATCAAAAGACAA	CTCGAG TTAGTAATCACTAGAATTTTC
U15	EHI_124550	CCCGGGATGAAAGATGGTTAT	CTCGAGTCAGATCAAAATGAT
U16	EHI_107170	CCCGGGATGATTGTATTTATATTGCT	CTCGAGTTAAAAGAATAAAAATGTGAGTGTAATAG
U18	EHI_176850	CCCGGGATGTCTGAAGATACTTCACCAATAAATGAG	CTCGAGTTATTTTTGATTTGTTGGTTGTTGAG
